# Phenology forcing model to estimate phenology shifting ability of extreme environmental events

**DOI:** 10.3389/fpls.2022.961335

**Published:** 2022-09-08

**Authors:** Aqeel Ahmad, Yujie Liu

**Affiliations:** ^1^Key Laboratory of Land Surface Pattern and Simulation, Institute of Geographic Sciences and Natural Resources Research, Chinese Academy of Sciences, Beijing, China; ^2^University of Chinese Academy of Sciences (UCAS), Beijing, China

**Keywords:** agriculture policy, climate change, crop modeling, maize, phenophase shifts, extreme environment

## Abstract

The current study considered the climate extreme index (*CEI*) values originated from extreme environmental events (EEEs) by following the National Oceanic and Atmospheric Administration (NOAA) guidelines. The EEEs were fractionated into six sub-categories (i.e., high temperature, low temperature, high precipitation, low precipitation, drought, and wind), and the combined impact of *CEIs* was utilized to develop an algorithm for the estimation of the phenology sensitivity index (*P*_*Si*_). Finally, the *CEIs*, and the *P*_*Si*_ were undergone the development of the phenology forcing (*P^F^*) model. The developed model showed a high sensitivity at the *CEI* value of as low as ≥1.0. Furthermore, the uncertainty index varied between 0.03 and 0.07, making a parabolic curvature at increasing *CEIs* (1.0–15.0). The current study precisely estimates the tendency of EEEs for phenology change. It will assist in policy-making and planning crop cultivation plans for achieving sustainable development goal 2 (SDG2) of the Food and Agriculture Organization (FAO).

## Introduction

Periodic events in the life of plants are very important for their survival and for the production of seeds to enter the next generation (Liu et al., 2021; Chen et al., 2022). Timely completion of the vegetative stage and a scheduled initiation of the reproductive (flowering) stage can warrant the production of healthy fruits and seeds (Yousaf et al., 2015; Liu et al., 2020a). This periodic occurrence of life events is called phenology. All the life events happening in a plant life cycle are studied under phenology, including seedling emergence, seedling growth stages, plant growth stages, initiation of the reproductive stage, transformation in reproductive stages, maturity, etc. These stages can be named differently based on easily different life events of different plants. But, the importance of phenology can never be over-emphasized in the successful completion of a plant life-cycle and to engender the next generation (Shafique et al., 2014; Ibrahim et al., 2017; Shah et al., 2021). The importance of phenology is amplified in agriculture as the farmers are interested in the timely maturity of the crops and the in-between events. A strictly scheduled growth pattern allows them to cultivate multiple crops round the year (Anjum et al., 2017; Yasin et al., 2019), and any deviation in the crop phenology can cause significant crop losses (Fatima et al., 2020).

Climate is the supreme factor affecting the plant life cycle and the timetable of their life events (Pan et al., 2019; Liu et al., 2020a). Generally, the plants grow in an open environment, and they have to face the environmental conditions directly without any protection shield. Therefore, plants are among the most exposed organisms to the environment (Bashir et al., 2016; Khan et al., 2019). On the other side, the environment has become unpredictable, and the frequent extreme events impact the plants the most (Ahmed et al., 2022). Although the climate has been changing over the last few centuries, the frequency of extreme events has been increased over the last few decades, and it continues to increase and challenge the life cycle of the agricultural crops (Abbas et al., 2017; Ahmed et al., 2018).

Generally, the phenology change or the phenophase shift is of two types, (i) transient phenology change (ii) permanent phenology change. Temporary shift in phenophase of the crop plant not persisting to the next year cultivated crops is categorized as transient phenology change (TPC). This type of phenology change is caused by extreme environmental factors under the survival threshold levels of the crop cultivars. Any permanent shift in the phenophase of a crop is categorized as a permanent phenology change (Visser and Both, 2005). It may happen either by (i) an extreme environmental factor exceeding the survival threshold levels of the crop cultivars or by (ii) periodic conterminous events of extreme climate for multiple years causing TPC. The frequent TPCs can influence the ecological timetable of local geographical communities, impacting the connected steps of the food chain, e.g., arthropods feeding on plants (Ettinger et al., 2021). Furthermore, frequent and consecutive phenology shifts can render farmers toward the selection of crop cultivars more tolerant to environmental extremes (Shafique et al., 2011; Khan et al., 2016).

Extreme environmental events (EEEs) that occur for a short period can cause significant crop losses and cause a complete failure of the cultivated crop. Several studies have reported the impact of climate change on crop phenology (Visser and Both, 2005; Ettinger et al., 2021; Liu et al., 2021). Similarly, now it is also a well-proven fact that the EEEs significantly impact crop phenology; however, the EEEs’ tendency to change crop phenology is yet to be disclosed. There is a large knowledge gap about the quantitative measurement of the phenology shift in plants resulting from the abrupt climate changes, as there is no research available to arithmetically estimate the tendency of EEEs for shifting phenophases. Furthermore, there is no reliable way to calculate the differential sensitivity of phenophases toward different types of extreme climates. Previously, researchers have been using some crop models in which the phenophase shift has been used as an input factor (Ahmad and Ashraf, 2016; Zhou et al., 2017; Czernecki et al., 2018; Zhao et al., 2018), e.g., univariate linear regression model, multiple linear regression model, etc. However, there was no model available to determine the potential of the extreme climate to advance or delay plant phenology. Therefore, we designed this study to develop a model to precisely measure the phenology shift tendency of the EEEs on agricultural crops in terms of phenology forcing index (*P^F^*). *P^F^* is an arithmetic representation of the perturbation tendency of an environmental factor to shift a phenophase of the plant either earlier or delayed. The plant population facing phenophase shift is termed the phenophase shift density (PSD), largely based on the type of phenophase and the type of crop plant determining phenology sensitivity. We have taken into account the phenology sensitivity during the algorithm development for this model. Therefore, the current model is able to provide the closest value of the phenology forcing index for the given EEEs. The model also considers six different classes of EEEs separately and has the capability to give them a share in determining the final values of *P^F^*. The study is a unique effort to arithmetically calculate the phenology shift tendency and play a key role in improving the accuracy of existing crop models. It will help to understand crop responses toward climate better and will assist the researchers in developing agriculture policies and future food security.

## Methodology

### Data used and algorithms development

Earth’s climate was considered stable before the industrial revolution occurred in 1750. Therefore, the change in climate and the climate forcing index are considered zero in 1750. Similarly, the phenology forcing index (*PFI*) is the arithmetic estimation of the potential of an extreme environmental event to shift a specific phenophase. The data were collected from the world weather & climate extremes archive of the World Meteorological Organization to perform the analyses. The calculation of *PFI* is carried out in a relative manner, considering it zero in 1750. The *PFI* is based on an aggregate set of conventional climate extreme indicators which, as described by the National Oceanic and Atmospheric Administration (NOAA)–National Centers for Environmental Information. NOAA climate database has enlisted six extreme climate factors derived from four types of environmental datasets; (i) extreme high temperature, (ii) extreme low temperature, (iii) extreme high precipitation, (iv) extreme low precipitation, (v) wind speed (storm, hurricane, etc.), and (iv) drought. All these six parameters possess their specific share in EEEs and have the tendency for phenology shift. We developed the algorithms to calculate the extent of each EEEs sub-category according to NOAA guidelines and combined them to get their collective impact.

### Description of extreme environmental events subclasses

The temperature was taken as the mean temperature value recorded in degrees Celsius (°C), while the precipitation was considered in millimeters (mm). Drought intensity was measured in terms of drought duration, based on the number of days without precipitation by following the palmer drought severity index (*PDSI*). At the same time, the wind speed was considered in kilometers per hour (km h^–1^) by neglecting its blowing direction. In this way, the extreme climate factors considered in this model development were the same as used in the calculation of the climate extreme index (*CEI*) by NOAA, while their detailed description is as follows:

1. Climate extreme index of high temperature (*CEI*_*HT*_) was calculated from the sum of (a) a percentage of the maximum temperatures below average high temperatures (*HT*_*Avr*_) and (b) a percentage of the maximum temperatures much above the *HT*_*Avr*_.
2. Climate extreme index of low temperature (*CEI*_*LT*_) was the sum of (a) the percentage of the minimum temperatures much below average low-temperature *LT*_*Avr*_ and (b) the percentage of the minimum temperatures much above *LT*_*Avr*_.
3. Climate extreme index of high precipitation (*CEI*_*HP*_) was the sum of (a) percentage of the high precipitation higher than the average high precipitation *HP*_*Avr*_ of the area and (b) percentage of the high precipitation lower than the *HP*_*Avr*_.
4. Climate extreme index of low precipitation (*CEI*_*LP*_) was the sum of (a) percentage of the low precipitation higher than the average low precipitation LP_*Avr*_ of the area and (b) percentage of the low precipitation lower than the LP_*Avr*_.
5. Climate extreme index of drought (*CEI*_*D*_) was calculated as the sum of (a) percentage of the days with no precipitation greater than an average number of days without precipitation (*DD*_*Avr*_) and (b) percentage of the days with no precipitation lesser than *DD*_*Avr*_.
6. Climate extreme index of wind speed (*CEI*_*W*_) was calculated as the sum of (a) percentage of the wind speed greater than average wind speed (*WS’*) and (b) percentage of the wind speed lesser than *WS’*.

In each case, the parameters were considered as much below and higher than the maximum and minimum environmental conditions. Furthermore, the representative values were screened as the tenth percentile of the period of record. Furthermore, the *CEI* values of each subclass of the extreme environment were summed up to calculate the total value of the climate extreme index (*CEI*).


$$C\,E\,I=C\,E\,I_{H\,T}\,C\,E\,I_{L\,T}\,C\,E\,I_{H\,P}\,C\,E\,I_{L\,P}\,C\,E\,I_{D}\,C\,E\,I_{w}$$

The zero value of *CEI* indicated that no fraction of the climatic parameter recorded had extreme conditions. However, the highest *CEI* value of 100 represented that the entire test area had extreme conditions throughout the recorded period.

The method devised by the National Centers for Environmental Information (NCEI) climate division precipitation and temperature databases were followed to calculate the *PDSI* (Karl et al., 1986). The *PDSI* categorized drought conditions in increasing order of intensity as near normal, mild to moderate, severe, or extreme for droughts and wet periods, depending upon the weeks passed under drought conditions, which fitted nicely into the *CEI* framework. Similarly, it had a large database for tropical storm and hurricane wind data, extracted from the National Hurricane Center’s North Atlantic Hurricane Database (HURDAT), which were added to the *CEI*.

### Impact of climate extreme index on phenology shift

The algorithms derived under NOAA guidelines assisted by NCEI based *PDSI* was used to calculate the impact of *CEI* on phenology shift using HURDAT based data in terms of phenology shift index (*P*_*Si*_). Meanwhile, the phenophase data was used to calculate the penology sensitivity (*S*_*CEI*_) by keeping in mind that all the plant species did not respond identically to the extreme environmental conditions in terms of phenophase shift. Taking into account the *P*_*Si*_ and *S*_*CEI*_ a phenology forcing index (*F^p^*) was devised as per the rate of climate extreme index with respect to phenology sensitivity. The details of the algorithms and the devised model were presented in the equations.

### Verification of phenology forcing index

High-resolution datasets about maize crops were procured from the HURDAT database and from the environmental data station of the institution to verify the results of the phenology forcing index. The datasets used in this study were of nClimGrid grade with a spatial resolution of 5 km and temporally distributed from 1979 to 2020. Furthermore, sample maize data of phenophase shift was used to compare the results of the model and the actual field conditions. All the procured datasets were undergone a forest analysis and a funnel plot to estimate the data deviation. The funnel test represented the reliability of the results. At the same time, the main characteristics of the funnel plot were the sample size and its statistical significance. The forest plot estimated the summary effect and analyzed the heterogeneity in the data components. The differential weight of the data components was represented with the square boxes, while horizontal lines represented the weight range. A starred square area showed the total variance of the components. The analysis was performed with a confidence interval (CI) of 95% along with the neutral point shown with a dashed line. The datasets were analyzed by PyCharm V:2021.1.1 × 64 (JetBrains; SRO) as an integrated development environment (IDE), as an extended platform of Python to draw time-course phenology shift plots. Furthermore, the graphical illustrations were presented to give a comparison between devised model and observed data.

## Results

The algorithm for the climate extreme index of high temperature was developed in the following form:


$$E\,H\,T=\left({\text{HT}}_{A\,v\,r}-\mathrm{HT}\right)\times \frac{H\,T}{100}$$

Whereas the deviation from the average of the tenth percentile of these values was considered as the climate *CEI*_*HT*_.


$$C\,E\,I_{H\,T}=\frac{\sum E\,H\,T^{10\,p}-E\,H\,T}{E\,H\,T}$$

Similarly, the algorithm representing the climate extreme index of low temperature was as follows:


$$E\,L\,T=\left({\text{LT}}_{A\,v\,r}-\mathrm{LT}\right)\times \frac{L\,T}{100}$$

However, the deviation from the average of the tenth percentile of these values was considered as the climate *CEI*_*LT*_.


$$C\,E\,I_{L\,T}=\frac{\sum E\,L\,T^{10\,p}-E\,L\,T}{E\,L\,T}$$

The following equation was the arithmetic representation of the algorithm for the climate extreme index of high precipitation.


$$E\,H\,P=\left({\text{HP}}_{A\,v\,r}-\mathrm{HP}\right)\times \frac{H\,P}{100}$$

While the deviation from the average of the tenth percentile of these values was considered as the climate *CEI*_*HP*_.


$$C\,E\,I_{H\,P}=\frac{\sum E\,H\,P^{10\,p}-E\,H\,P}{E\,H\,P}$$

The algorithm represented the climate extreme index of low precipitation was as follows.


$$E\,L\,P=\left({\text{LP}}_{A\,v\,r}-\mathrm{LP}\right)\times \frac{L\,P}{100}$$

However, a deviation from the average of the tenth percentile of these values was considered as the climate *CEI*_*LT*_.


$$C\,E\,I_{L\,P}=\frac{\sum E\,L\,P^{10\,p}-E\,L\,P}{E\,L\,P}$$

Climate extreme index based on drought developed the following equation.


$$E\,D=\left({\text{DD}}_{A\,v\,r}-\mathrm{DD}\right)\times \frac{D\,D}{100}$$

And a deviation from the average of the tenth percentile of these values was considered as the climate *CEI*_*LT*_.


$$C\,E\,I_{D}=\frac{\sum E\,D^{10\,p}-E\,D}{E\,D}$$

The climate extreme index of wind was processed to develop the following algorithm.

$E\,W=\left(W\,S-W\,S^{\prime }\right)=\frac{W\,S^{\prime }}{100}$ And a deviation from the average of the tenth percentile of these values was considered as the climate *CEI*_*W*_.


$$C\,E\,I_{w}=\frac{\sum E\,W^{10\,p}-E\,W}{E\,W}$$

### Impact of climate extreme index on phenology shift

After the successful development of the algorithms for all sub-categories of EEEs, the second step was to estimate the relation between the *CEI* and phenology shift, which was developed in the form of a phenology shift index as described in the following equation.


$$P_{S\,i}=\frac{{\left(\sum △\,P\right)}^{n_{p}}}{100\times N_{p}}$$

Where ∑△*P*is the sum of all the phenology shifts in a test phenophases; and the *N_p_* is the total number of phenophases studied.

The detailed equation to calculate phenology sensitivity was as follows.


$$S_{H\,T}=\frac{\left({\left(\sum △\,P\right)}^{n_{p}}/100\times N_{p}\right)}{C\,E\,I}$$

The algorithm devised for the calculation of phenology forcing index and its elaborated equation was as follows.


$$F^{p}=C\,E\,I/S_{C\,E\,I}$$

$$F^{p}=\frac{C\,E\,I^{2}\times N_{p}\times 100}{{\left(\sum △\,P\right)}^{n_{p}}}$$

### Verification with a sample data

**FIGURE 1:**
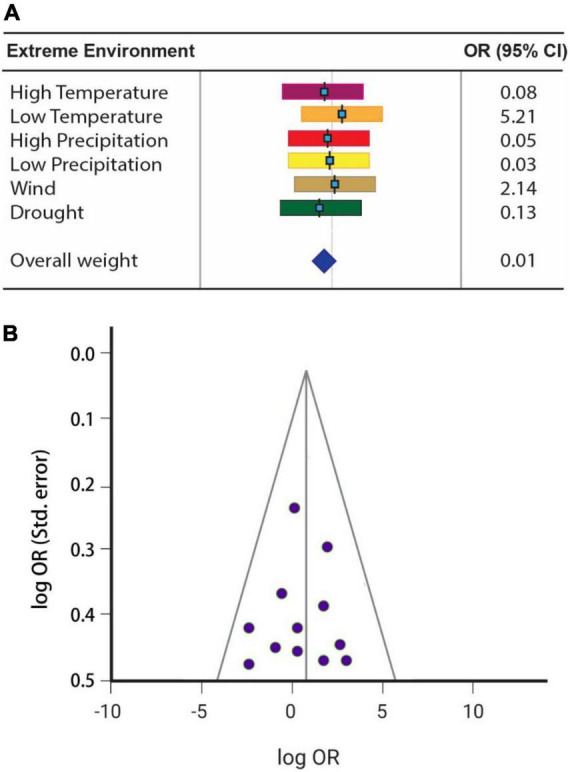
Forest plot of the procured data for verifying phenology forcing model **(A)** calculated at a confidence interval of 95%. The funnel plot represents the reliability of the data components based on the log OR-log odds ratio **(B)**.

The maximum heterogeneity among the datasets was 5.21, while the maximum inclination of the datasets representing perturbation in phenology shift was recorded at 0.13. The average data cover calculated in the analysis was 0.01, as shown in the forest plot (Figure 1A). Besides, no dataset was found with significant deviation compromising the reliability of the results. Almost all the procured datasets were ranged under the limits as calculated by 95% confidence interval, with maximum data points in the funnel’s first quadrate (bottom side). However, each of the second and third quadrates had one data point (Figure 1B).

**FIGURE 2:**
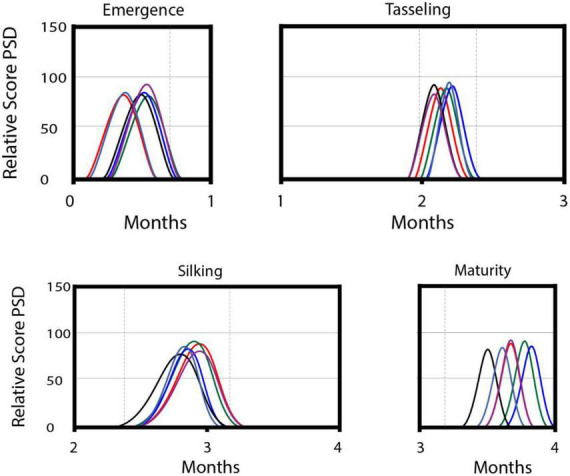
An illustration of the impact of phenology forcing on phenology shift of four phenophases of maize crop. Time considered in the study has been considered in months and mentioned on the *x*-axis, while the score of phenology shift density (PSD) has been mentioned on the *y*-axis. The role of each sub-class of EEEs has been represented with differentially colored lines; high temperature (red); low temperature (green); high precipitation (blue) low precipitation (purple); drought (black); wind (gray).

The phenophase shift modeled by the phenology forcing index revealed the highest phenology shift at both the terminal stages of a plant’s life, i.e., seedling emergence and maturity. These stages showed the highest phenology shift of >15% of their total duration under the influence of almost every sub-class of EEEs. Drought and high temperature were the two parameters that shifted the plant phenophases and changed the rate of PSD per unit time duration. Low temperature prolonged the seedling emergence and silking phenophases up to 32 and 17% of their individual duration, respectively. However, drought had the opposite impact and shortened the plant life by increasing the value of PSD within a short interval of time (Figure 2).

### Model verification

**FIGURE 3:**
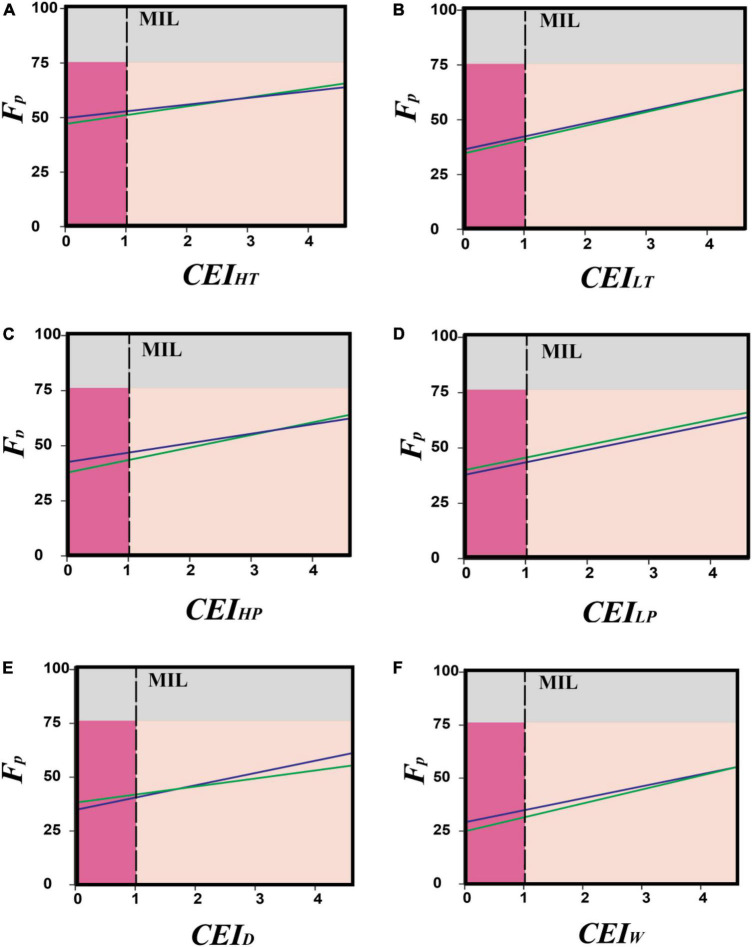
A phenology forcing comparison between the observed data and the serial increment data of six extreme environmental categories, i.e., high temperature **(A)**, low temperature **(B)**, high precipitation **(C)**, low precipitation **(D)**, drought **(E)**, and wind speed **(F)**. climate extreme index values have been plotted on the *x*-axis, and the phenology forcing index value has been mentioned on the *y*-axis. Minimum impact limit (MIL).

The comparison between the sample maize data (observed data) and a data series obtained thorough serial increment showed almost parallel elevations of phenology forcing index in most cases. Especially in the case of low precipitation, there was no point in interception between the two lines representing both of the data classes. Although the observed data values were initially a little less, the area of this difference was lower than the minimum impact limits of the model, which were set at *CEI* ≥ 1.0. The EEEs of high precipitation and drought showed the highest deviation among all environmental sub-categories; however, their deviation pattern was not identical to each other. The phenological forcing due to drought did not deviate initially, but its value increased with the increasing climate extreme index value (*CEI*_*D*_). However, in the case of high precipitation, the phenological forcing values were closer to the model values with the increasing *CEI*_*HP*_. An identical trend (just like high precipitation) was recorded in the case of wind speed (Figure 3).

**FIGURE 4:**
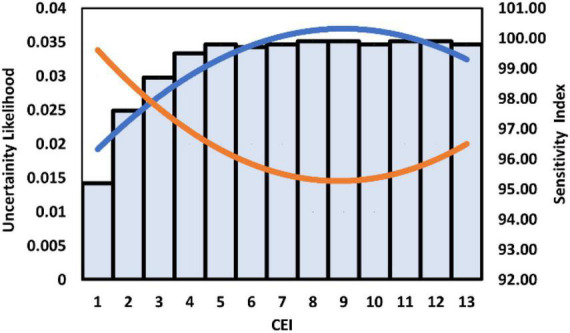
Uncertainty and sensitivity analyses of the phenology forcing model. A trend line colored blue represents the sensitivity index values as mentioned on the right vertical axis. While the orange-colored trend line shows the uncertainty likelihood in the model as mentioned on the left vertical axis. The climate extreme index (*CEI*) is mentioned on the *x*-axis.

The sensitivity index of the devised model was increased from *CEI* values of 1–4; however, it got stable at later stages. The sensitivity index was recorded as 95.2 at the very initial *CEI* value of 1.0, and then it was increased up to 99.5 at the *CEI* value of 4.0. At later stages, it showed slight variability between 99.5 and 99.8 at different *CEI* values. However, in the case of uncertainty analysis, the first four *CEI* values showed an opposite trend with remarkably decreasing uncertainty up to *CEI* = 4.0. However, during *CEI* 4–12, it remained stable and again showed an increase between *CEI* 13–15 (Figure 4).

## Discussion

Although climate change is perilous for crop cultivation plans and patterns, it is common to observe that sudden and intense changes in climate could cause more damage to crops than gradual slow changes. Therefore, climate extreme index- *CEI* was first introduced in early 1996 (Gleason et al., 2008; Liu et al., 2020b; Chen et al., 2022) to summarize the multidimensional and multivariate combinations of environmental conditions. The *CEI* helped the inexperienced persons to understand the overall environmental impact and to draw the impact on agricultural crops. The *CEI* concept was so interesting and wide in its application that it was adopted in various fields of research related to the environment. The current study is an advancement to the *CEI* concept as it introduces the impact of *CEI* on crop phenology. Furthermore, it is the first effort to apply *CEI* values for crop modeling. Therefore, the current investigation occupies a unique position in understanding the impact of extreme climate on agricultural crops.

The most important aspect of the current investigation is the consideration of all six sub-categories separately in the development of algorithms. Generally, climate studies related to crop cultivation are restricted to climate change, and they don’t consider the abrupt and short-term changes in the climate, the extreme environmental events (Athar et al., 2021; Chen et al., 2022). However, these extreme environmental events greatly impact crop cultivation and food security. The current investigation has not only been extended to the EEEs, but it also developed separate algorithms for all six sub-categories of the EEEs, and then calculated their combined impact on plants in terms of phenology forcing. It is an implication of the NOAA guidelines to calculate EEEs impact on plant phenology. By using this phenology forcing model, researchers and environmental institutes could estimate an extended shift in crop phenophase and its possible effects on ecological balances and the food chain.

Most of the time, climate change is not a simple interaction between two variables. We have to consider a mesh of interlinked parameters while dealing with climate change (Yasin et al., 2018; Ahmad et al., 2021). For example, the carbon cycle is a multifaceted parameter that impacts the global environment (Fariduddin et al., 2006; Mehmood et al., 2018). It is interlinked with all the major parts of every ecosystem of this earth and continuously affects all types of life. All life control lies in carbon emissions and sequestration processes occurring in ecosystems. Its emission into the environment causes carbon fertilization, positively impacting plant growth and promoting carbon sequestration. At the same time, it is contributing to greenhouse gases and causing global warming (Tariq et al., 2021). Due to the complexity of the interlinked factors with the carbon cycle, its overall impact on the environment is difficult to be determined precisely (Rehman et al., 2020; Li et al., 2021; Abbas et al., 2022). Therefore, most of the time, we get only stochastic measures to estimate environmental factors, which is a major hurdle in achieving sustainable development goal 2 (SDG2) of the Food and Agriculture Organization—FAO (Ahmad et al., 2014; Khan et al., 2015). Moreover, we cannot estimate the precise nutritional distribution in the food chain due to unreliable estimation of the phenology shift of food crops (Amoroso, 2018). Additionally, the increased global warming has boosted the frequency and intensity of EEEs, making it more complex to understand the environment and its impact on agricultural crops. Considering all of these facts, there is a continuous need to develop improved models for a better understanding of plants’ responses toward EEEs. The study contributes a unique model to better understand the climate impact on crop phenology.

Climate change has the ability to alter the energy balance in a multivariate way, and this ability of energy change alterations is calculated in terms of climate forcing (Foster et al., 2017). It corresponds to energy transformation and energy flow from radiation to carbon sequestration, air pressure dynamics, aerosols mechanics, etc. (Foster et al., 2017; Zhao et al., 2019). However, there is no previous mechanism available to understand the tendency of climate change to change crop phenology (Liu et al., 2021; Zhou et al., 2021). The unpredictable phenology change also resulted in a less-reliable estimation of the interrelations among ecological components. Due to this, the overall impact of extreme environmental events could not be fully explained. Being an extremely important aspect of a plant’s life, it was a prominent gap in scientific knowledge that the current investigation has fulfilled. Now, the researchers can easily estimate the ability of the extreme climate to affect the life cycle events of a targeted crop and can devise better crop cultivation policies. Furthermore, the impact of EEEs on the ecological relationships of the plants and their dependent species can be correctly estimated.

Researchers must revise the environment-related model repeatedly with multiple datasets and at different geographical locations (Ahmad et al., 2018; Tan et al., 2021). Most of the time, this task is performed on a set of observed data in comparison to the set algorithms in the model. An example of this revision was the modifications in the *CEI* index in 2003 when it was improved with experimental and observed datasets (Gleason et al., 2008; Zhao et al., 2017; Yang et al., 2021). Researchers have to collect the observed data from multiple years and compare it with *CEI* simulations to generate a more reliable algorithm applicable to more diverse geographic regions and climatic conditions. A similar type of activity has also been performed with the current devised model, in which the developed algorithm has been compared with observed datasets to verify its output. Although, there will always be a need for more improvements by running its algorithms on spatially and temporally more distributed datasets. But, the initial verification process results are satisfactory and close to the natural output or phenology shift. Moreover, the sensitivity and uncertainty analyses have also proved that the model consists of a highly reliable set of algorithms and can detect the phenology shift from the fraction of *CEI* value.

Due to complexities in the EEEs and poor understanding of how they affect agricultural crops, we cannot use the primitive models due to their high error values and increased uncertainty (Wang et al., 2005; Liu et al., 2020a). The main reason for their unsuitability for EEEs is that they were not built for environmental extremes (Pan et al., 2019; Zhang et al., 2021). The researchers made their efforts to develop new models with the least error in the predicted environmental change and related factors. However, there was still a wide gap between the actual impact of EEEs dynamics in an ecosystem and its modeled values. This gap has been a cause of serious uncertainties in predicting EEEs impact on the crops hampering the experts in designing future agriculture policies with substantial confidence. The current study has precisely synchronized the EEEs with the phenophases of the maize crop to narrow this gap of uncertainties and bring more confidence in the predictive operations of agriculture policy-making and crop cultivation. Moreover, the designed model can be practiced in the global fields to get the intended benefits.

## Data availability statement

The original contributions presented in this study are included in the article/supplementary material, further inquiries can be directed to the corresponding author.

## Author contributions

YL conceived the idea. AA executed the project, collected, and analyzed the data. YL and AA wrote and revised the manuscript. Both authors contributed to the article and approved the submitted version.
